# Ten big challenges ahead

**DOI:** 10.1186/1471-2334-14-S2-S1

**Published:** 2014-05-23

**Authors:** Mark Wainberg

**Affiliations:** 1 McGill AIDS Center, Montreal, Quebec, Canada

## 

1. Can We Cure AIDS? This is an intense area of investigation at the current time with much focus on the activation of latently infected cells within reservoirs by such agents as histone deacetylase inhibitors. However, there is concern about some of these approaches due to considerations of possible long-term toxicities involving the activating agents as well as questions about whether it will be possible to activate all latently infected cells or not.

2. Can We Provide High Quality HIV Drugs to All People at Need Regardless of Ability to Pay? At present, people living in developing countries often receive sub-standard therapy, with drugs such as Raltegravir being available only for second-line therapy. So, when will our best new drug, ie Dolutegravir, become available in resource-limited settings?

3. How Will We Pay? There is a real risk that worldwide assistance programmes will run out of funds.

4. Will Treatment as Prevention Work in the Real World? Hopefully, the answer to this question will be Yes, but we have already seen that other prevention strategies have experienced difficulties when employed outside the context of a controlled clinical trial.

5. Will Drug Resistance Compromise All Prevention Strategies? Perhaps it is too early for HIV drug resistance virologists to retire. The answer to this question will be key and only time will provide an answer.

6. When Will We Have a HIV Vaccine? There is a very limited rate of success in the development of vaccines to all forms of sexually transmitted disease, with the obvious exception being Human papilloma virus. We should be more honest about this.

7. Are Microbicides Still in the Game? Clearly, they would probably never be as popular as PREP, if it worked, or as Treatment as Prevention.

8. Do PrEP and Intermittent PrEP Have a Chance? Certainly, these are strategies that should be effective when used together with counselling.

9. What if HIV Failed to Develop Resistance to a New Drug in First-Line Therapy? If this were ever to happen, it would change our thinking on how to prevent HIV transmission in a dramatic way. For example, the use of such a drug should potentially eliminate future new HIV transmissions. How significant is it that no-one who has received Dolutegravir in first-line therapy has yet developed resistance against it or is this only a matter of time?

10. What is the Impact of Global Warming on Infectious Diseases and Will Future Dengue Epidemics Surpass the HIV Pandemic in Incidence, Morbidity, and Mortality? The range of the mosquitos that transmit West Nile virus has already expanded to significant extent. Can Dengue be far behind?

